# Semaglutide-associated hyposalivation: A report of case series

**DOI:** 10.1097/MD.0000000000036730

**Published:** 2023-12-29

**Authors:** Hani Haytham Mawardi, Soulafa Adnan Almazrooa, Siraj Ahmed Dakhil, Ali Anwar Aboalola, Thamer Abdulmohsin Al-Ghalib, Rawah Talal Eshky, Adham Abdulmajeed Niyazi, Mohammed Haytham Mawardi

**Affiliations:** a Department of Oral and Diagnostic Sciences, King Abdul-Aziz University – Faculty of Dentistry, Jeddah, Saudi Arabia; b Department of Endodontics, King Abdul-Aziz University – Faculty of Dentistry, Jeddah, Saudi Arabia; c Department of Maxillofacial Surgery and Diagnostic Sciences, King Saud Bin Abdulaziz University for Health Sciences, Riyadh, Saudi Arabia; d King Abdullah International Medical Research Center, Riyadh, Saudi Arabia; e Dental Services, Ministry of the National Guard-Health Affairs, Riyadh, Saudi Arabia; f Department of Prosthodontics, Batterjee Dental College, Jeddah, Saudi Arabia; g Department of Preventive Dental Sciences, Taibah University – College of Dentistry, Medina, Saudi Arabia; h Department of Medicine, King Faisal Specialist Hospital and Research Center, Jeddah, Saudi Arabia.

**Keywords:** frothy saliva, hyposalivation, semaglutide, semaglutide-induced hyposalivation, xerostomia

## Abstract

**Rationale:**

Obesity and diabetes of different types are considered global health risks with rising prevalence. In addition to low-calorie diet and daily exercise, several treatment options have been introduced to help patient in needs. Semaglutide (Ozempic) is one popular agent, which attracted the attention of both physicians and patients due to its positive outcome in improving glucose control and weight loss. However, no reports on the effect of semaglutide use on the oral cavity and specifically xerostomia are available in the literature. We are reporting 3 cases for patients who were using semaglutide and developed secondary xerostomia.

**Patient concerns:**

Three female patients with median age of 34 (range 27–46) presented to the oral medicine clinic with chief complaint of xerostomia. All patients were overweight with a mean body mass index of 35.6 (range 35–37) and have been using semaglutide for weight loss for a mean duration of 11.3 weeks (range 6–16).

**Diagnoses:**

All 3 patients had severe dryness in the mouth with minimal frothy saliva with mean modified Schirmer test of 9 mL at 3 minutes (range 8–10 mL). Following exclusion of other possible underlying medical problems, the diagnosis of semaglutide-induced hyposalivation was given to all patients.

**Interventions:**

The patients’ management varied between discontinuation of the drug, the use of pilocarpine, and conservative symptomatic management.

**Outcomes:**

The patients resumed acceptable salivary flow.

**Lessons:**

We are reporting for the first time hyposalivation associated with the use of semaglutide. Further prospective, larger studies are warranted to confirm these findings.

## 1. Introduction

Obesity and diabetes of different types are considered global health risks with rising prevalence.^[1]^ Due to their significant impact on human health, glucose control and weight loss became the main goal for health care practitioners using all available tools.^[1]^ In addition to low-calorie diet and daily exercise, several treatment options have been introduced to help patient in needs.^[2]^ These ranges from different categories of medications to surgical options with variable indications, risks, and prognosis.

Semaglutide (Ozempic) is one popular agent, which attracted the attention of both physicians and patients due to its positive outcome in improving glucose control and weight loss.^[3]^ It is a subcutaneous, potent weekly injection acting as a glucagon-like peptide-1 (GLP-1) receptor agonists resulting in weight loss independent of body mass index (BMI) and gastrointestinal complications.^[4]^ Semaglutide has a safe profile in general, with common reported toxicities including hypoglycemia, pancreatitis, gallbladder stones and less commonly pancreatic and thyroid cancer.^[5]^ However, no reports on the effect of semaglutide use on the oral cavity and specifically xerostomia or hyposalivation are available in the literature.

We are reporting 3 cases for patients who were using semaglutide and developed secondary hyposalivation. We believe this is the first report to describe this potential toxicity associated with semaglutide use, which may have a significant impact on quality of life.

## 2. Case description

### 2.1. Case 1

**Figure 1.**
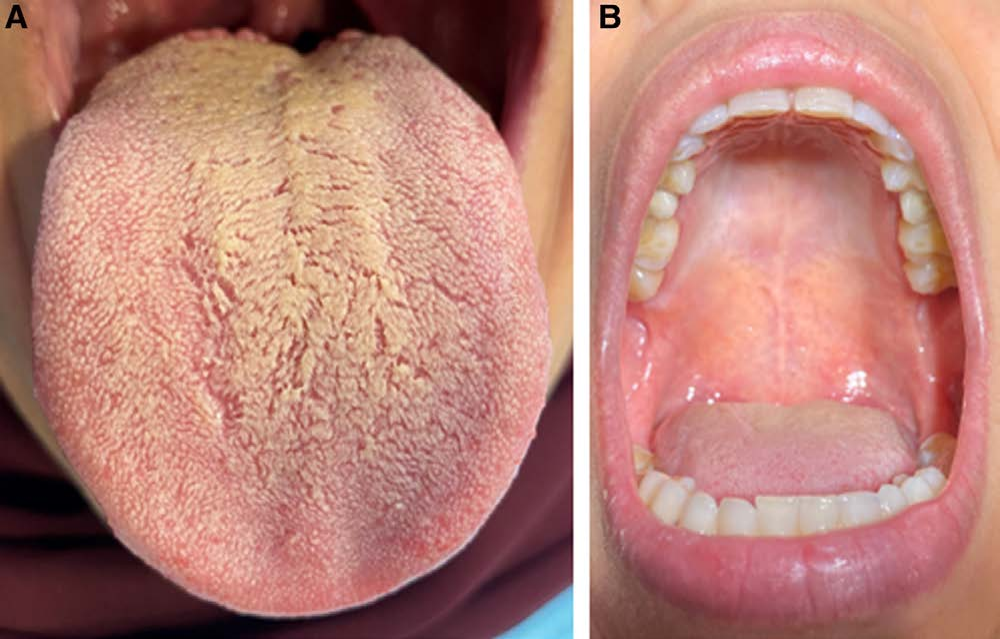
Clinical photo for Case 1 showing (A) dry and hairy dorsal tongue; and (B) hard palate with complete lack of saliva.

A 46-year-old female with history of rheumatoid arthritis and overweight (BMI of 35) currently on methotrexate (for total of 3 years) and semaglutide for weight loss (0.5 mg/subcutaneous weekly injection for total of 12 weeks) presented to the oral medicine clinic. The patient’s chief complaint was xerostomia and coated tongue for the past 8 weeks. No dryness in the eye was reported by the patient. The patient reports drinking 1.5 to 2 L of water, and 1 cup of tea daily. On clinical examination, the patient had white, non-scrapable coated tongue. The mouth was dry, with sticky mucosa and minimal frothy saliva (Fig. 1). In order to measure the unstimulated salivary flow, modified Schirmer test was used and showed 10 mL at 3 minutes.^[6]^ Part of the patient clinical evaluation, Sjogren syndrome screening tests were conducted, and all came back within normal limit. In addition, recent hemoglobin A1c (HBA1C) was 5.9. Based on the provided history and clinical examination, the patient was diagnosed with medication-induced hyposalivation associated with semaglutide. The patient was referred to the endocrinology clinic, and semaglutide was discontinued. At 4-weeks follow-up visit, the patient resumed normal salivary flow with modified Schirmer test showing 30 mL at 3 minutes.

### 2.2. Case 2

**Figure 2.**
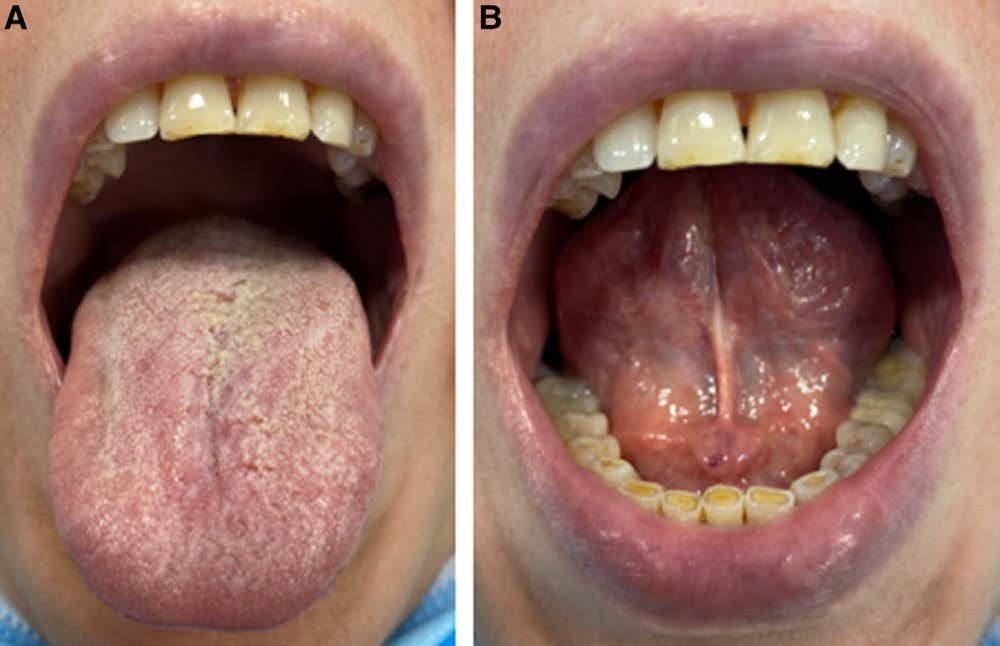
Clinical photo for Case 2 showing dry (A) dorsal tongue; and (B) floor of the mouth with complete lack of saliva.

A 27-year-old female with history of overweight (BMI of 37) currently on semaglutide for weight loss (0.5 mg/subcutaneous weekly injection for total of 16 weeks) presented to the oral medicine clinic with a chief complaint of xerostomia and dry lips for the past 12 weeks. Patient denied any eye dryness. The patient reports drinking 1.3 to 1.7 L of water and 2 cups of coffee daily. On clinical examination, the patient had dry, chapped lips. The oral mucosa was dry with sticky surfaces and minimal frothy saliva (Fig. 2). The modified Schirmer test was used to measure the unstimulated saliva and showed 9 mL at 3 minutes. Sjogren syndrome screening tests were conducted, and all came back within normal limit. In addition, recent HBA1C was 6. Based on the provided history and clinical examination, the patient was diagnosed with medication-induced hyposalivation associated with semaglutide. Following consultation with the patient’s physician, she was instructed to continue semaglutide for medical purposes. To manage the patient’s chief complaint, she was started on pilocarpine 5 mg/day. At 4-weeks follow-up visit, the patient resumed an acceptable salivary flow with modified Schirmer test showing 25 mL at 3 minutes.

### 2.3. Case 3

**Figure 3.**
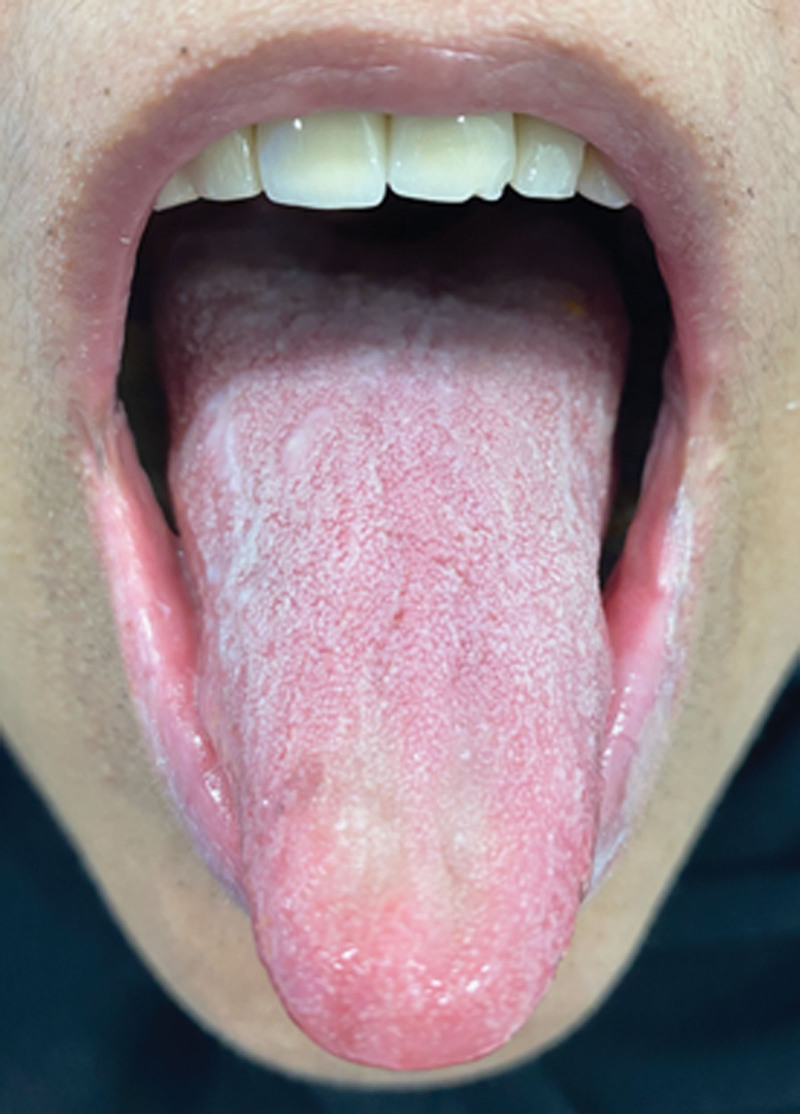
Clinical photo for Case 3 showing dry dorsal tongue and lips with frothy and thick saliva.

A 34-year-old female with history of overweight (BMI of 35) currently on semaglutide for weight loss (0.5 mg/subcutaneous weekly injection for total of 6 weeks) presented to the oral medicine clinic with a chief complaint of xerostomia and halitosis for the past 2 weeks. Patient denied any eye dryness. The patient reports drinking 2.5 L of water with 1 cup of coffee daily and continues to feel thirsty. On clinical examination, the patient had dry mouth, with minimal frothy saliva (Fig. 3). The modified Schirmer test was used to measure the unstimulated saliva and showed 8 mL at 3 minutes. Sjogren syndrome screening tests were conducted, and all came back within normal limit. In addition, recent HBA1C was 5.9. Based on the provided history and clinical examination, the patient was diagnosed with medication-induced hyposalivation associated with semaglutide. The patient chose to continue being on semaglutide until she reaches her target weight. At 16-weeks follow-up visit, the patient presented for clinical evaluation, and she had an acceptable salivary flow with modified Schirmer test showing 27 mL at 3 minutes.

## 3. Discussion

Xerostomia is a common complaint defined as the subjective report of dry mouth with significant impact on patients’ quality of life including speech, chewing, and swallowing.^[7]^ It is a common problem with variable severity with estimated prevalence among general population ranging between 5.5% and 46%.^[7]^ Often, xerostomia is commonly associated with hyposalivation which is the objective reduction of saliva in the mouth.^[7]^ The saliva can be frothy, and thick. The mucosa can look shiny and dry. It can stick to the wooden tongue depressor and/or the dentists’ gloves. Based on the literature, the average stimulated salivary flow rate ranges between 1.5 and 2 mL/min and unstimulated salivary flow rate averages between 0.3 and 0.4 mL/min.^[8]^ Common causes for hyposalivation include the use of certain medications (e.g., antidepressants, antihypertensives, and steroid inhalers), history of radiation therapy to the head and neck area and immune-related conditions such as Sjogren syndrome.^[8]^ Chronic hyposalivation are likely to have a negative impact on the oral cavity and may result in rampant caries, oral fungal infections, and halitosis.^[9]^

This report discusses 3 cases presenting with severe hyposalivation noted following the use of semaglutide commonly used for glucose control, obesity as well as neurodegenerative diseases such as Parkinson and Alzheimer.^[10]^ Semaglutide was initially approved by the US Food and Drug Administration in 2017 for the treatment of type 2 diabetes.^[11]^ Later in 2020, the approval was extended for cardiovascular risk reduction in adults with type 2 diabetes and heart disease available in oral and subcutaneous forms.^[11]^ It is an effective GLP-1 receptor agonist which is a prominent hormone responsible for glucoregulatory process, and plays a major role in digestion by delaying gastric emptying and appetite suppression.^[10]^ In addition to the intestine, GLP-1 is also expressed in the brain affecting water intake, neurogenesis satiety center, stress reaction, and energy homeostasis.^[12]^ Other reported mechanism of action for semaglutide include natriuretic and diuretic properties, reduction in renal hypoxia as well as reduction in renal angiotensin II.^[13]^ In general, semaglutide is well tolerated and considered safe based on multiple clinical trials. The reported toxicities are mostly mild and include nausea, diarrhea, headache, and urinary tract infection.^[5]^

The initial differential diagnosis for hyposalivation reported in the 3 cases was dehydration and low water intake which was within the lower range of normal for both patients. However, considering the severity and duration of reported symptoms, dehydration was less likely to be the underlying cause. In addition, no history of radiation therapy to the head and neck area was reported by either patient. Another differential diagnosis considered was systemic disease-related hyposalivation (i.e., diabetes and Sjogren syndrome). However, both patients denied any dryness in the eye and had normal lab values for Sjogren panel (i.e., rheumatoid factor, anti-Ro, anti-La, and antinuclear antibodies).^[14]^ In addition, no classical signs or positive laboratory results for diabetes were reported by both patients. As one of the patients was on methotrexate for rheumatoid arthritis, a literature search was conducted and no reports on the association with hyposalivation was found.^[15]^

As semaglutide was a common agent used by both subjects for weight loss, it was considered as a possible underlying cause for multiple reasons. First, the known mechanism of action and body water loss properties for semaglutide may explain the risk of hyposalivation in patients on semaglutide. Second, chronic diarrhea is a common side effect reported for semaglutide which could lead to total body dehydration.^[5]^ Third, Yabe et al^[16]^ reported nasopharyngitis as a common adverse with semaglutide use which may facilitate mouth breathing and dry mouth. Fourth, the treatment duration with semaglutide reported by patients and onset of hyposalivation may support the diagnosis of semaglutide-associated hyposalivation for all cases. One interesting observation in this report is the onset of hyposalivation in which it was noted 4 weeks after starting semaglutide.

As of today, no reports have linked the use of semaglutide with hyposalivation. In addition to semaglutide, albiglutide, exenatide, liraglutide, lixisenatide, and dulaglutide are all GLP-1 receptor agonists available in the market with no reports of hyposalivation or even xerostomia as an expected adverse effect have been published.^[17]^ It could be reasonable to start educating patients about to start on semaglutide on the risk of hyposalivation and the need for close follow up with their dentists for early detection and management.

## 4. Conclusion

We are reporting for the first-time hyposalivation associated with the use of semaglutide. Although this finding is based on 3 reported cases, this observation shed light on possible oral complication associated with semaglutide long-term use. Further prospective, larger studies are warranted to confirm these findings.

## 5. Patients’ perspective

All 3 patients presented to the clinic with xerostomia which affected their quality of life and lead them to the oral medicine clinic to address this complaint. They were involved in evaluating the severity of their symptoms along with the treating physician’s consultation which affected the choice to discontinue the drug or continue using it and treating the symptoms.

## Author contributions

**Conceptualization:** Hani Haytham Mawardi, Soulafa Adnan Almazrooa, Siraj Ahmed Dakhil, Adham Abdulmajeed Niyazi, Mohammed Haytham Mawardi.

**Data curation:** Hani Haytham Mawardi.

**Investigation:** Mohammed Haytham Mawardi.

**Methodology:** Hani Haytham Mawardi, Siraj Ahmed Dakhil, Ali Anwar Aboalola, Adham Abdulmajeed Niyazi, Mohammed Haytham Mawardi.

**Project administration:** Ali Anwar Aboalola, Thamer Abdulmohsin Al-Ghalib, Rawah Talal Eshky.

**Resources:** Hani Haytham Mawardi.

**Supervision:** Hani Haytham Mawardi, Soulafa Adnan Almazrooa, Adham Abdulmajeed Niyazi, Mohammed Haytham Mawardi.

**Validation:** Siraj Ahmed Dakhil, Ali Anwar Aboalola, Adham Abdulmajeed Niyazi, Mohammed Haytham Mawardi.

**Visualization:** Soulafa Adnan Almazrooa, Siraj Ahmed Dakhil, Ali Anwar Aboalola, Thamer Abdulmohsin Al-Ghalib, Rawah Talal Eshky.

**Writing – original draft:** Hani Haytham Mawardi, Ali Anwar Aboalola, Thamer Abdulmohsin Al-Ghalib, Rawah Talal Eshky, Adham Abdulmajeed Niyazi, Mohammed Haytham Mawardi.

**Writing—review & editing:** Hani Haytham Mawardi, Soulafa Adnan Almazrooa, Siraj Ahmed Dakhil, Thamer Abdulmohsin Al-Ghalib, Rawah Talal Eshky, Adham Abdulmajeed Niyazi.
